# Hyperscanning: A Valid Method to Study Neural Inter-brain Underpinnings of Social Interaction

**DOI:** 10.3389/fnhum.2020.00039

**Published:** 2020-02-28

**Authors:** Artur Czeszumski, Sara Eustergerling, Anne Lang, David Menrath, Michael Gerstenberger, Susanne Schuberth, Felix Schreiber, Zadkiel Zuluaga Rendon, Peter König

**Affiliations:** ^1^Institute of Cognitive Science, Universität Osnabrück, Osnabrück, Germany; ^2^Institut für Neurophysiologie und Pathophysiologie, Universitätsklinikum Hamburg-Eppendorf, Hamburg, Germany

**Keywords:** hyperscanning, social cognition, joint action, EEG, MEG, fMRI, fNIRS, social interactions

## Abstract

Social interactions are a crucial part of human life. Understanding the neural underpinnings of social interactions is a challenging task that the hyperscanning method has been trying to tackle over the last two decades. Here, we review the existing literature and evaluate the current state of the hyperscanning method. We review the type of methods (fMRI, M/EEG, and fNIRS) that are used to measure brain activity from more than one participant simultaneously and weigh their pros and cons for hyperscanning. Further, we discuss different types of analyses that are used to estimate brain networks and synchronization. Lastly, we present results of hyperscanning studies in the context of different cognitive functions and their relations to social interactions. All in all, we aim to comprehensively present methods, analyses, and results from the last 20 years of hyperscanning research.

## Introduction

The importance of social interaction for the development and maintenance of the human self was already highlighted in Greek philosophy and has been discussed ever since. Nevertheless, the field of cognitive neuroscience has only started to investigate brain activity during social interaction in the last decades. Typically, only the brain of one of the involved participants and thus only one part of the dyadic or group interaction was recorded at a time. The insight such experiments may provide is therefore limited. To examine social interactions as a whole, the idea of hyperscanning, i.e., measuring the activity of multiple brains simultaneously, has originated. The significant advantage of this technique is that it allows the investigation of real-time dynamics between two or more interacting brains (Hari and Kujala, 2009; Hari et al., 2013). In contrast to classic experimental paradigms that measure the brain activity of single participants during social interaction, simultaneously measuring the brain activity of several interacting participants allows for the investigation of intra- and inter-brain neural relations (Schilbach et al., 2013). The hyperscanning techniques thus offer a new approach to account for the complexity of joint action, i.e., its spontaneity, reciprocity, and multimodality, which constitutes a big challenge for its neuroscientific examination.

In the current paper, we have reviewed existing literature and evaluated the current state of the hyperscanning method. We performed extensive literature research to identify the most critical peer-reviewed studies that used hyperscanning as a method to investigate human social cognition. In our review, we have had two primary goals. First, we reviewed the methods and types of analysis that are used in the hyperscanning field. Second, we reviewed cognitive functions and their neural underpinnings that are investigated with the hyperscanning method.

## Type of Methods

In the last century, a large variety of methods to measure brain activity have been developed. The most popular ways to measure brain activity used in the cognitive neuroscience field are Electroencephalography (EEG) (Luck and Hillyard, 1994), Magnetoencephalography (MEG) (Baillet, 2017), Functional magnetic resonance imaging (fMRI) (Eisenberger, 2003), and functional near-infrared spectroscopy (fNIRS) (Ferrari and Quaresima, 2012). Each of these have their advantages and disadvantages, which can help us to further understand different brain functions. Primarily, when focused on Hyperscanning, their specific assets, like temporal and spatial resolution as well as mobility, are of value. We have reviewed here all of these in the context of Hyperscanning research.

### fMRI

Functional magnetic resonance imaging (fMRI) is a method that indirectly measures neural brain activity. Namely, it measures it by detecting changes associated with blood flow, which is the blood-oxygen-level-dependent (BOLD) contrast (Glover, 2011). Since the last decade of the twentieth century, fMRI has become one of the most popular methods used in cognitive neuroscience. Its most important advantage is the spatial resolution. Standard fMRI scanners estimate brain activity with a spatial resolution of 3 mm, and specialized instruments push the limits toward the sub-millimeter range.

**Figure 1:**
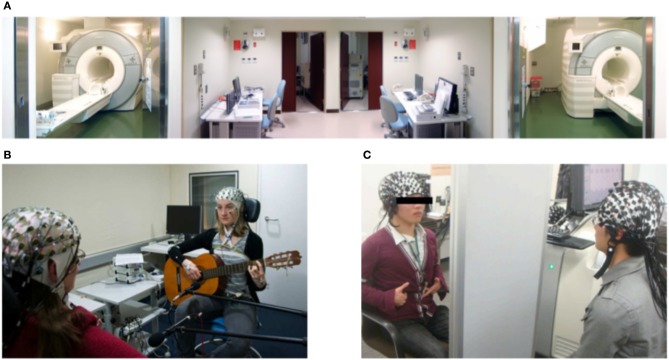
Neuroimaging methods used in Hyperscanning. **(A)** From Koike et al. (2019). View of the dual fMRI facility used to study mutual gaze. **(B)** From Acquadro et al. (2016). EEG measurement of two guitar players. **(C)** From Osaka et al. (2015). fNIRS set up used to study cooperative singing. All parts reproduced/adapted under CC licenses.

In comparison to other methods discussed below, it is the best method to determine where in the brain something has happened. Additionally, it is the non-invasive method of choice for measuring deep brain structures. However, because it uses blood flow to estimate neural activity, its temporal resolution does not compare to M/EEG (Glover, 2011). Moreover, to measure the BOLD signal, participants are required to stay stable in a laying position within a scanner (Figure 1A). This low mobility of the experimental tools makes it not suitable for investigating social interactions in naturalistic and ecologically valid setups. Despite low mobility, the first-ever hyperscanning study was an fMRI study. Montague (2002) performed a successful feasibility study to link participants in two scanners. To tackle the problem that occurs when two scanners are required to complete a study, King-Casas (2005) conducted a study using scanners in Texas and California linked via the Internet. Afterward, other studies were performed in facilities that possessed two scanners; however, until now, only a few studies have tried to investigate social interaction with the fMRI hyperscanning method (Tomlin, 2006; Saito et al., 2010; Schippers et al., 2010; Tanabe et al., 2012; Tomlin et al., 2013; Spiegelhalder et al., 2014; Koike et al., 2016, 2019; Abe et al., 2019). One reason for that might be difficulty in creating experimental paradigms that involve interaction between participants without movement and communication. Another reason might be that the complexity of fMRI data requires the development of new types of analysis that are suitable to answer questions about between-brain relations. It is sensible to say that the value of each of these studies is excellent, and more studies are required because fMRI has a good spatial resolution. Furthermore, this method could be of great value if combined with EEG to surmount poor temporal resolution (Koike et al., 2015).

### EEG/MEG

One of the oldest methods to measure activity in the brain is electroencephalography (EEG). In comparison to fMRI and fNIRS, it measures neural activity directly by recording electrical activity with the use of electrodes placed on the scalp (Figure 1B). Therefore, it is not dependent on blood oxygenation, and its temporal resolution is higher than other methods (Michel and Brunet, 2019). However, because electrodes are placed on the scalp, it is best suited for investigating the cerebral cortex and not deep brain structures. Classically, EEG was considered a low mobility tool because it required strict control of the movements and surroundings of participants, which limits it to the lab environment. However, in the last years, the development of new technologies has allowed for improvement in mobility by creating mobile EEG systems (Melnik et al., 2017). Such systems are a great tool to study social interactions. Even though fMRI was the first method used to perform a hyperscanning study, it is EEG that is currently the most common method used to conduct hyperscanning experiments. Its popularity comes from its most important advantage, temporal resolution. Studies of social interaction that unfold on a fast scale require a method that is sensitive to it. Until now, only EEG could account for changes in neural processing on a millisecond scale while two or more humans perform an interactive task together. The high temporal resolution allows for a more precise and different type of between-brain analysis. Another advantage of EEG for hyperscanning studies is that it is easier to measure more than just two heads at the same time, as demonstrated by Dikker et al. (2017). The relatively low price of EEG systems and the availability of mobile systems are key advantages. Early EEG hyperscanning research was conducted in the lab with full control of the environment and traditional paradigms (Babiloni et al., 2007a,b). However, with further developments, more interactive and naturalistic paradigms, like playing guitars (Lindenberger et al., 2009) or romantic kissing (Müller and Lindenberger, 2014), were proven to be feasible. In recent years, another technology, which can be combined with EEG, was developed and implemented to use in research. Namely, virtual reality (VR) (Ehinger et al., 2014; Oliveira et al., 2016; Cipresso et al., 2018) is becoming more and more present in the scientific community. It allows for creating naturalistic paradigms that are fully controlled by the experimenter. This, in combination with the EEG, might be a great tool to study social interactions.

It is worth mentioning that magnetoencephalography (MEG), a method with similar characteristic to EEG but lower mobility, was also proven to be feasible for hyperscanning measurements (Baess et al., 2012; Zhdanov et al., 2015), and it has so far been used in a study that combined it with EEG to study verbal interactions (Ahn et al., 2018). Moreover, this method was also utilized to study the interaction between mothers and children (Hirata et al., 2014; Levy et al., 2017), speaker-listener roles during natural conversation (Mandel et al., 2016), and hand kinematics in leaders and followers (Zhou et al., 2016). Recently, Boto et al. (2018) developed a mobile MEG system. Therefore, we can expect more MEG hyperscanning studies in upcoming years.

### fNIRS

The last neuroimaging method that we have reviewed is functional near-infrared spectroscopy (fNIRS). Similarly to fMRI, it measures brain activity indirectly and uses the contrast between oxygenated and de-oxygenated hemoglobin, and similarly to EEG, it can best measure superficial brain areas with a low spatial resolution (1 cm) (Scholkmann et al., 2013) (Figure 1C). Moreover, its temporal resolution is lower than that of EEG and varies between 0.1 and 1 s (Quaresima and Ferrari, 2019). Despite these limitations, fNIRS is widely used in cognitive neuroscience for its mobility and resistance to motion artifacts. In comparison to other methods discussed here, the signal measured with fNIRS is not strongly influenced by the movement of participants. This feature allows for creating experimental paradigms that resemble real-life situations more closely than classic studies. In the case of studying social interaction that involves actions from participants, it is a critical feature that is required. The first hyperscanning fNIRS study was conducted by Funane et al. (2011) and used a simple tapping synchronization task to investigate the coherence of neural activity between two brains. Since then, many researchers adopted hyperscanning fNIRS in various types of paradigms to study social interactions (Scholkmann et al., 2013). One particularly interesting study was conducted by Nozawa et al. (2016). It involved groups of participants (four) tested in a naturalistic setting (cooperative communication). Furthermore, a recently developed fNIRS system for babies allows for investigating brain functions related to parent-child interaction (Reindl et al., 2018). Such experiments are proof of the concept that studying neural between-brain underpinnings is feasible, and it brings new insight into the understanding of human cognition.

## Type of Analysis

The analysis and interpretation of hyperscanning data is a challenging task. First, an intra-brain type of analysis has to be adjusted to inter-brain data; alternatively, new types of analysis have to be developed. Second, it is challenging to separate inter-brain relations related to identical stimuli presented to both participants from relations that represent between-brain networks (Burgess, 2013). For the case of correlation, this involves the calculation of partial or semi-partial correlation coefficients. Similar adjustments might be done to other measures. An alternative approach compares real participant pairs with randomly selected pairs and a permutation analysis (e.g., Bilek et al., 2015). The randomly selected pairs show only the coupling due to the direct joint stimulation. Deducting this effect from the coupling observed in the actually paired subjects uncovers the coupling of brain activity due to the genuine interaction of the partners. Overall, this is a demanding topic, and it requires precise specification of the scientific question addressed.

Furthermore, while discussing between-brain coupling measures, it is essential to mention the framework proposed by Hasson et al. (2012). It suggests that inter-brain couplings are crucial for building a shared social world. This framework builds upon research that focused on between brain couplings without the hyperscanning method. Namely, Hasson et al. (2004) presented videos to individual participants in the fMRI scanner and further analyzed between brain couplings (between all participants) related to different sections of the movie. Further, Stephens et al. (2010) used the same method to study speakers and listeners (scanning one speaker and many individual listeners to investigate the relation between the speaker and the listener). These studies were crucial for the development of the hyperscanning field and contributed to the understanding of between-brain couplings. He investigated single subjects; there are only randomly selected pairs, yet the similarities are interesting and give insight into brain functioning. Thus, the assumed control of randomly selected pairs can demonstrate interesting and insightful similarities (coupling) between participants.

The types of analyses applied to hyperscanning data can be separated into different categories. There are various coupling measures, correlation and dependence analyses, graph theory measures, and the analysis of information flow. In this section, we have discussed all these types of analyses in sequence.

### Coupling/Connectivity Measures

**Figure 2:**
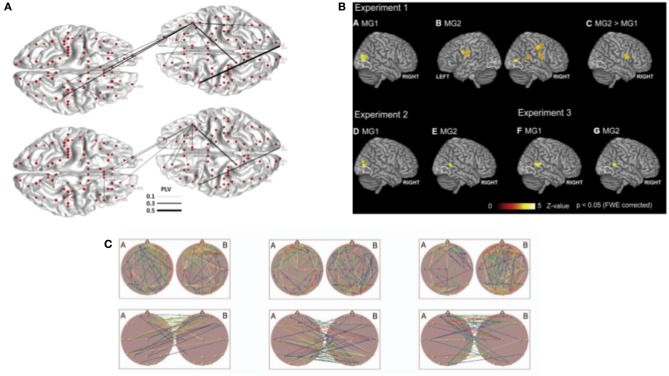
Analysis methods used in hyperscanning to investigate between brain relationships. **(A)** From Yun et al. (2012). Phase synchrony was used as a coupling measure to investigate between-brain connections in implicit coordination task. Topography of the phase synchrony (PLV) between different regions of interest of two participants are presented for theta (4–7.5 Hz) and beta (12–30 Hz) oscillations. **(B)** From Koike et al. (2016). Between-brain synchronization estimated with correlation. **(C)** From Müller et al. (2013). Brain topography maps illustrating significant connection within and between the brains. Example of graph theory measures applied to analyze synchronization during musical improvisation on the guitar. All parts reproduced/adapted under CC licenses.

The most common methods to estimate the strength of coupling/connectivity between brains have previously been used to study single brains. They are based on second-order measures calculated in the Fourier domain. They differ in the technical details of combining different frequencies and the kind of normalization. That is, like the phase-locking value (PLV), the phase lag index (PLI), or phase coherence have been adopted to estimate between-brain couplings. PLV measures how two signals (in case of hyperscanning coming from two different brains) are phase-locked in the observed time window. PLV is equal to 1 when phases are perfectly synchronized in a specific frequency and to 0 when they are unsynchronized. This measure was used in multiple EEG hyperscanning studies. They investigated cortical synchronization while two participants tried to imitate their hand (Dumas et al., 2010, 2011) or finger movements (Yun et al., 2012) (Figure 2A) during a coordinated time estimation task (Mu et al., 2016), during speaking and listening (Pérez et al., 2017), and during a cooperative decision-making task (Hu et al., 2018). Another similar measure, also related to phase synchronization, PLI, was used in studies investigating coordinated behavior in guitar players (Lindenberger et al., 2009; Sänger et al., 2012) and also in a verbal interaction task with the use of both EEG and MEG (Ahn et al., 2018). PLV and PLI are similar measures; however, it was pointed out that PLV suffers the common source problem, and PLI does not (Aydore et al., 2013). However, for hyperscanning research, where sources are separated between brains, these measures should give the same results. Phase coherence is another method of estimating cortical synchronization within or between brains that are related to the phase of neural oscillations. It is a measure of similarity between two signals, and there is more than one way of quantifying it. Different variations of phase coherence were used in hyperscanning experiments (for detailed differences between different phase measures we recommend Thatcher, 2012). Notably, studies mentioned above investigated guitar players (Lindenberger et al., 2009; Sänger et al., 2012, 2013; Müller et al., 2013) as well as romantic partners while kissing (Müller and Lindenberger, 2014). Moreover, the latter study also estimated cross-frequency couplings between brains.

Wavelet transform coherence (WTC) is a related method to measure the coherence of two signals. It was developed to analyze the geophysical time series (Grinsted et al., 2004). However, it finds its application also in neuroscience, especially in analyzing fNIRS hyperscanning studies. Since one of the first fNIRS studies (Cui et al., 2012), it was used in the following experiments. As it is the most common method that is used to analyze inter-brain synchrony with fNIRS, it is also the most common analysis method within all hyperscanning studies. WTC was used to estimate inter-brain synchrony in paradigms studying action monitoring (Dommer et al., 2012), cooperative and competitive behaviors (Cui et al., 2012; Osaka et al., 2014; Cheng et al., 2015; Liu et al., 2016b; Wang et al., 2019), imitation (Holper et al., 2012), verbal communication (Jiang et al., 2012, 2015; Nozawa et al., 2016), non-verbal communication (Osaka et al., 2015; Hirsch et al., 2017), decision making (Tang et al., 2016; Zhang et al., 2017), coordination (Hu et al., 2017; Ikeda et al., 2017; Pan et al., 2017) , mother-child problem solving (Nguyen et al., 2019), and teaching/learning behaviors (Pan et al., 2018).

All coupling/connectivity measures mentioned in this section are measures of similarity between two neural signals coming from different brains. This similarity is interpreted as synchrony between brains in these studies. Moreover, the similarity is estimated with different methods and is always referred to as inter-brain synchrony. Such simplification of many analysis methods to address synchronization may lead to wrong interpretations of results and creates a wrong view of coherence between studies. The hyperscanning field should develop terms to distinguish between different measures of inter-brain synchrony and methods used to estimate it.

### Correlation and Dependence Analysis

Another way of measuring synchrony between brains is by estimating the correlation between signals coming from two brains. Within the hyperscanning literature, we found different types of correlation measures applied to EEG, fNIRS, and fMRI data. Due to the low temporal resolution of fMRI, coupling measures discussed in the previous chapter could be used only in the very-low-frequency range, which is not typically associated with information processing in the brain. Therefore, the relation between two measured brains is often estimated with the use of linear dependence. It is not the BOLD signal itself that is used for correlation analysis, but regression model coefficients are representing activations in different tasks. These types of analyses were applied in research investigating mutual gaze, shared attention, and cooperation in the joint force production task (Saito et al., 2010; Koike et al., 2016, 2019; Abe et al., 2019) (Figure 2B). Correlations found in these studies were interpreted as neural synchronization between brains.

Further, two studies focused on verbal communication between participants and used correlation of BOLD activity to predict the flow of information between the sender and perceiver (Anders et al., 2011) and synchronization of brain activity between interlocutors (Spiegelhalder et al., 2014). To further extend the dependence analysis, cross-correlation in combination with ICA decomposition of the BOLD signal was used in studies focused on joint attention in participants with borderline personality disorder and healthy participants (Bilek et al., 2015, 2017). There, the cross-correlation between two brain signals was interpreted as information flow.

Correlation measures are also applied to EEG hyperscanning data. Namely, we identified studies using correlation as a measure of between brain synchronization in different paradigms. Moreover, different aspects of EEG signals were used for correlation analysis. Correlation between different frequencies (theta and alpha) was used to investigate the coordination of speech rhythm (Kawasaki et al., 2013) as well as differences between interactions between strangers and couples in alpha, beta, and gamma (Kinreich et al., 2017). Furthermore, the total independence analysis (Wen et al., 2012) was used in a study that investigated between brain synchronization in a class environment on a group of students (Dikker et al., 2017; Bevilacqua et al., 2019). This analysis was used to predict classroom dynamics and engagement.

Lastly, two fNIRS experiments applied correlation analysis to estimate synchrony between brains in tasks that required cooperation or competition between participants (Funane et al., 2011; Liu et al., 2015).

Similarly to coupling measures, the correlation and dependency analysis leave ambiguity about how to relate results from different studies due to a variety of methods applied to estimate the phenomenon of synchronization.

### Graph Theory Measures

Between-brain networks can also be quantified with graph theory measures. Up to today, there are only studies that used graph theory measures on EEG hyperscanning data. Graph theory measures focus on different aspects of between brain networks. Within existing hyperscanning literature, we found studies that focused on links between brains and modularity of networks while participants performed the decision-making task (De Vico Fallani et al., 2010). Moreover, different graph theory measures were used to investigate between-brain networks in guitar players. Small-worldness of between-brain networks was enhanced during musical coordination (Sänger et al., 2012), and the topology of between-brain networks was dependent on frequency and was more regular at higher frequencies (Müller et al., 2013) (Figure 2C). Additionally, the directionality of between-brain networks was used to predict leaders and followers in guitar players (Sänger et al., 2013). In another study, the dimensionality of between brain networks was investigated in combat cooperation tasks (Dodel et al., 2011). All mentioned measures suggested that neural synchrony can be estimated with graph theory measures and that these measures extend our understanding of between brain networks. Few studies mentioned here give great insights into understanding neural dynamics between brains. We believe that graph theory measures are a great tool to account for the complexity of inter-brain relations. Measures like modularity, small-worldness, and directionality are bringing a new perspective into understanding neural underpinnings of dynamic social interactions. More studies should explore these measures. Additionally, more data-driven methods to define network properties are becoming more popular and can find their application in hyperscanning as well (Sporns, 2018).

### Information Flow

Apart from synchrony, similarity, or network properties, one can focus hyperscanning analysis on the flow of information from one brain to another. Such studies require estimating causal links between brains. Methods that are used to determine such causal links are Granger Causality and its equivalent in a frequency domain Partial Directed Coherence (PDC). In the EEG hyperscanning literature, these methods were applied to estimate links between brains of cooperating pilots (Astolfi et al., 2011, 2012), and results suggested that causal links are stronger during increased cooperative behavior. Similarly, increased causal links between the brains of participants were found in cooperative and altruistic behaviors in decision-making tasks (De Vico Fallani et al., 2010; Ciaramidaro et al., 2018). Furthermore, one fMRI and one fNIRS study focused on causal links between brains. Schippers et al. (2010) studied such links in gesture communication with the use of fMRI and Pan et al. (2017) used fNIRS to explore causal relationships between brains of cooperating lovers. The casual links between brains can be estimated with methods that we discussed here; however, the important question of what the neural substrates that allow for information flow between brains are is yet to be answered. It is critical to understand the difference between information flow and synchronized neural activity between brains due to identical sensory input. This problem is often not addressed and left for readers to wonder how to disentangle both. Future research should focus on this aspect.

Taken together, in this section, we reviewed different methods and types of analysis that are used in the hyperscanning field. A variety of techniques and analysis suggests that hyperscanning is a new and valuable part of the cognitive neuroscience field. However, in many cases, the advantages and disadvantages of a specific method are not that obvious. Further, at least in part, we consider the growing variety of techniques used as exploratory, and it has to be investigated whether they relate to the same set of physiological processes.

## Cognitive Functions

### Coordination and Synchronization

The investigation of interpersonal coordination of actions that includes mutual entrainment or synchronization is one of the field's most suited for hyperscanning. Simultaneously measuring the brain activity of interacting subjects allows for real-time access to the reciprocal coupling of neural processes that enable interpersonal movement synchronization within a millisecond time scale. Experimental paradigms are addressing the connection between interpersonal neural dynamics and behavioral synchronization span from minimalistic button-pressing tasks to complex naturalistic settings like joint music playing. In minimalistic tasks, different parameters, such as visual contact, feedback, and mode of synchronization (in-phase vs. antiphase), can be manipulated easily. Additionally, several studies have compared the degree of behavioral synchronization between human-human and human-computer (metronome) couples (Konvalinka et al., 2014; Mu et al., 2016; Hu et al., 2017) in order to extract the social aspect of the interaction. Such setups enable the examination of various aspects of action coordination and synchronization while controlling the effects of a shared sensory environment.

One conventional paradigm is used to study coordinated behavior and its neural underpinnings and requires participants to perform only one temporally synchronized button press after a predefined or self-time interval has passed. As a result of this, better performance was related to higher inter-brain coherence in frontal areas as well as to stronger social connectedness in the dyad (Funane et al., 2011; Mu et al., 2016; Hu et al., 2017; Pan et al., 2017).

Another paradigm used continuous tapping or finger/hand movements, allowing for additional insight into the time course and the dynamics of synchronization. Tognoli et al. (2007) found that the spontaneous transition from uncoordinated to coordinated rhythmic movements under vision went along with specific EEG rhythms in the alpha mu range at right centro-parietal sides. Dumas et al. (2010) took a between-brain approach, using the Phase Locking Value (PLV) across a variety of different frequency bands. He found that right parietal alpha mu oscillations were significantly more coupled in periods of spontaneous synchronization. Both results point toward the relevance of these patterns for the mirror neuron system. A similar paradigm also investigating alpha-band activity was used by Naeem et al. (2012a,b). However, they did not replicate Tognoli's approach but focused on broader frequency bands in the mu range in different coordination contexts (intrinsic, in-phase, and antiphase), suggesting functional discrimination of the lower (8–10 Hz) and upper (10–12 Hz) mu band (Naeem et al., 2012a). While the former seems to reflect general attentional processes, the latter is modulated by task and hemisphere: in the left hemisphere, the top mu band is present during imitation, while in the right hemisphere, it is involved in perceptual-motor discrimination. Based on this, the authors suggest a right hemispheric circuit that modulates the way the actions of others are processed concerning the desired coordination mode (Naeem et al., 2012b). In another study that focused on the directionality of interaction, the subject associated as the leader showed a characteristic suppression of frontal alpha activity, possibly representing enhanced cognitive control and planning (Konvalinka et al., 2014). Manipulating the neural synchronization between the participants with tACS, two studies directly explored the impact of phase-coupled neural oscillations on behavioral performance. Results indicated that in-phase beta but not alpha or theta stimulation across the respective motor cortices facilitated tapping entrainment (Novembre et al., 2017; Szymanski et al., 2017a). However, it was not yet shown whether this effect could be replicated in EEG studies.

In contrast to such minimalistic experimental setups, several studies applied hyperscanning in more cognitively demanding and also more naturalistic settings. Recording two subjects interacting in a finger-tapping imitation task, Holper et al. (2012) observed increased functional connectivity between two interacting brains. Social aspects modulated even unconscious fingertip movement synchronization: Yun et al. (2012) found that after having cooperated in an induced imitation task, the patterns of unconscious finger movement across two subjects became more synchronized. On a neural level, this change went along with increased theta and beta band phase locking across different regions, including the inferior frontal gyrus (IFG), anterior cingulate cortex (ACC), and ventromedial prefrontal cortex (vmPFC). In this context, the researchers associated the observed activity patterns with implicit social processing.

In a cognitively more demanding task, Lindenberger et al. (2009) investigated pairs of guitarists playing a melody together. Similarly, they reported that coordinated actions between the subjects involved oscillatory couplings between the two brains. During coordinated play onset, they found significantly increased phase synchronization between the two brains, primarily over frontal-central connections. The synchronization was exceptionally robust in low-frequency ranges, between 0.5 and 7.5 Hz, with a maximum in the theta frequency at 3.3 Hz. This result contradicted previously mentioned studies that primarily reported dominant alpha synchronization during interpersonal action coordination. The authors, however, noted that the observed couplings might merely reflect similarities in the temporal structure of the individual's perception and action. Accordingly, it is still not clear whether the neural coupling causes the effective movement coordination between the pairs. Rather than reporting specific brain areas and frequency bands, the studies mentioned above suggest that inter-brain connectivity through interpersonally coupled brain oscillations facilitates complex interpersonal action coordination.

Social aspects of action coordination, i.e., the influence of social connectedness and social character traits on synchronization performance, is another topic where multiple brain recordings provide new valuable insight. On a hormonal level, Mu et al. (2016) could show that oxytocin, intranasally administered, significantly facilitates neural synchronization in the alpha band and thus effectively supports movement coordination. Addressing the influence of the social connectedness between pairs, Pan et al. (2017) compared the performance of lovers to strangers and friends in a simple coordination task. Between the lover's brains, they indeed found a significantly increased synchronization. More specifically, they reported that the right frontoparietal network was involved in romantic processing and social cognition. At the same time, lovers also showed a significant increase in coordination performances. Since oxytocin is strongly associated with social bonding, especially in romantic relations, this result supports Mu et al. (2016), indicating the human hormone's facilitating effect on interpersonal action coordination. Applying the same simple interaction task, Hu et al. (2017) found a correlation between the prosocial inclination of the subjects and their respective inter-brain synchronization. All in all, these studies supported the evidence that social traits and the ability to synchronize in interpersonal coordination are strongly connected.

The current hyperscanning research on synchronization and coordination reports neural (synchronization) effects in various areas and frequency bands, although exploring very similar tasks. There are two groups of findings: the first group reports inter-subject neural couplings in frontal and parietal regions that are associated with better action synchronization (theta, alpha, and beta frequency). The second group of findings focuses on mechanisms that are not coupled across individuals but correspond to how a single brain processes incoming stimuli in a coordination context (Tognoli et al., 2007; Naeem et al., 2012a,b; Konvalinka et al., 2014). Interestingly, these within-brain effects were also reported at frontal and centro-parietal sides in the alpha range. They all indicate that interpersonal action synchronization is accompanied by neuronal coupling of primarily frontal and centro-parietal areas in lower frequencies. However, concerning prominent frequency bands related with movement synchronization, the reported results do not seem to be conclusive: while some effects were mainly within the alpha (10–12 Hz) and beta (~20 Hz) range, others specifically excluded the alpha range and instead emphasized a synchronization in the theta frequency (Lindenberger et al., 2009; Yun et al., 2012; Novembre et al., 2017). Such differences in reported effects of activity in different frequencies have to be addressed in future research.

Apart from this, prosocial character traits, such as the social connectedness of the dyads, influenced the effectiveness of synchronization. However, since many of the mentioned studies had fewer than 10 pairs of subjects, more work is needed to ensure and replicate the results.

### Music

Musical performances offer attractive experimental conditions since such performances combine intrapersonal action coordination and interpersonal action synchronization as well as continuous interaction. The advantages of musical settings for hyperscanning experiments are reviewed by Acquadro et al. (2016). A variety of experimental paradigms allow for the investigation of different aspects of the interaction.

To investigate that inter-brain synchronization during an interaction is not only present due to the perception of the same ecological situation, researchers assign roles to the participants to investigate if complementary roles induce asymmetric patterns of brain synchronization. Sänger et al. (2012) investigated interpersonal action coordination using EEG hyperscanning of musical leader-follower duets playing a two-voiced piece of music repeatedly. They reported within-brain phase-locking modulated by the assigned role as well as extended within- and between-brain phase coherence during phases of high musical coordination. Because of the complementary voices of the piece, the phase coherence occurs in a situation where the action and perception of the partners are not equal. Further, graph theory analyses show the presence of hyperbrain network structures. Later analysis of the same data by Sänger et al. (2013) allows for investigation of the directionality of functional connectivity between the two brains. Results show directionality as a function of the musical roles. Pan et al. (2018) recorded brain activity of learner-instructor dyads during the acquisition of two songs using one of two learning methods (part learning vs. whole learning). The study recorded fNIRS data of bilateral fronto-temporoparietal regions. Across the part-learning group, they report interpersonal brain synchronization during the learning periods, which was even able to predict the learning performance. Furthermore, Granger causality analyses show coupling directionality from instructor to learner during a particular learning phase (teaching phase). The absence of interpersonal brain synchronization correlations in the whole learning group speaks against it as a mechanism of pure shared perception since both learning groups received equal sensory input and performed comparable actions. Synchronous oscillations are a present mechanism in leader-follower musical joint action tasks, and the asynchronous nature of these signals gives rise to inter-brain synchrony partly as a mechanism of interactive task performance.

Other experimental designs investigate music without assigned roles, as this is the case in many musical contexts. While some experiments use existing music pieces, others engage in freer musical interactions like non-notated parts of songs or even improvisation. Novembre et al. (2016) used the structured properties of sheet music to manipulate familiarity and behavioral interpersonal synchronization during joint piano playing. With dyads of amateur piano players performing passages of two-voiced joint playing with congruent and incongruent instructions for a later tempo change as well as alternating knowledge about the complementary voice, they reported significant correlations between alpha suppression and congruent vs. incongruent tempo instructions in the case of the pianist being familiar with both voices of the passage. The authors concluded with the idea of alpha oscillations as neural processes regulating the balance between self-other integration and segregation, modulated by the compatibility of internal knowledge and external environmental information during joint action. After verifying EEG as a suitable method for hyperscanning in a musical context, Babiloni et al. (2011) used a hyperscanning paradigm to investigate empathy inside ensembles of musicians, playing a piece together (Babiloni et al., 2012). Alpha desynchronization in the right Brodmann area 44/45 during a video observation of their performance is positively correlated to the results of the Empathy Quotient Test score. Müller et al. (2013) investigated musical improvisation in dyads of guitarists. They analyzed intra- and inter-brain synchrony during either a phase of joint improvisation or phases where one guitarist improvised while the other listened. They reported high-frequency intra-brain connections as well as lower frequency inter-brain connections. Guitarists playing alone showed stronger out-strength than the listening guitarist in the beta range; this difference was not present during joint improvisation. Osaka et al. (2014) compared fNIRS inter-brain coherence of participants during cooperative humming of a song with or without eye contact and single humming. Results indicated enhanced wavelet transform coherence inside the right inferior frontal cortex (IFC) during the non-face-to-face condition. A further study, Osaka et al. (2015), compared the inter-brain synchrony between dyads humming or singing a song, again with or without visual contact, solo and joint. The left IFC showed increased synchronization for joint singing or humming, irrespective of the visual condition, while the right IFC showed increased synchronization specific to joint humming. The absence of synchrony in solo performances and aligned pseudo-pairs suggests the involvement of bilateral IFC in (musical) cooperation tasks. Inter-brain synchrony is a present mechanism even in more unstructured musical interactions, acting as a marker of interpersonal action coordination. Furthermore, experimental musical setups can be used for methodological analyses, as in the case of Zamm et al. (2018).

Altogether, the present results confirm musical paradigms as highly coordinative situations generating the ability to observe inter-brain synchrony as a mechanism of interpersonal action coordination with a high potential for future research.

### Emotion and Affect

Emotional regulation and affect play a crucial role in various forms of social interactions, such as the willingness to undertake joint actions with peers (Lopes et al., 2005) or in different types of prosocial behavior (Twenge et al., 2007). Neuroscientific studies measuring emotion and affect based only on one participant's data lack the inter-brain connections among areas that might be involved in social behavior. In order to fill this gap, hyperscanning allows for recording inter-brain activity on emotions' onset and the simultaneous responses of interacting people.

To address the emotional component in social exchanges, several hyperscanning paradigms have been applied. Among these, setups have involved facial communication of affect (Anders et al., 2011), mother-child interactions (Hirata et al., 2014; Levy et al., 2017), and goal-seeking tasks involving cooperation and competition conditions (Pan et al., 2017). Nonetheless, due to the complexity of the setups (i.e., Hirata et al., 2014), hyperscanning studies have scarcely focused on the role of emotional regulation during joint actions (Ciaramidaro et al., 2018), leading in most cases to merely exploratory designs and vague hypotheses (Balconi and Vanutelli, 2017).

As an example of emotion processing during goal-oriented tasks, Hu et al. (2017) studied the prosocial behavior of dyads while performing a task in which participants performed coordinated and independent tasks across several trials. The authors found synchronized inter-brain activity only under the coordination task in the left middle frontal cortex (LMFC). This area has been commonly associated with memory, response inhibition, and people recognition during social interactions. Besides, Ciaramidaro et al. (2018) performed a study in which participants had the opportunity to distribute a quantity with a partner. A third participant (the observer) would judge the fairness of the distribution and decide whether to punish or not the participant who acted out the distribution. The dyads of participants involved in the exchange were composed of either human-human or computer-human. EEG data revealed higher inter-brain coherence of theta, alpha, and beta bands in the human-human condition between the observer and the receiver when the latter's action was rated as “hyper-unfair.” Additionally, synchronous activity was also robust for PC-human fair interactions where the human participant received a fair reward. As the authors stated, situations with high emotional impact showed higher inter-brain synchronization.

In another experiment, Anders et al. (2011) investigated the emotional communication in romantic partners by observing the flow of information in male participants for emotional states perform by their female counterparts. They suggested that the neural activity of the perceiving partner can be successfully predicted from the neural activity of the sender's brain. This shared activation could only be found in dyads comprised of romantic partners but not in dyads of the sender and another participant different from her romantic couple. This finding suggested the development of reinforced neural paths present among sexual partners with highly emotional bindings.

Finally, some studies on emotions in social interaction have addressed the simultaneous measurement of inter-brain activity between mother and child. Levy et al. (2017), for instance, used a hyperscanning MEG setup to measure the brain-to-brain activity of mother-child dyads by exposing them to video recordings of themselves performing positive and conflictual interactions. They found gamma activity in the superior temporal sulcus (STS) in interactions with behavioral synchrony (i.e., positive interactions). STS has been amply linked to social cognition, the theory of mind, and mirroring behavior. In the same line, Hirata et al. (2014) developed a hyperscanning MEG device that enables the mother and child to see each other's facial expressions during brain activity measurement. Although mainly of an explorative kind, these studies comprised a relevant background as pioneers of experimental designs to account for emotional interaction in hyperscanning setups.

Although not extensive, these studies highlight the moderator effect that the emotional component has in inter-brain activity in two scenarios. First, the closer the relationships between participants, the higher the inter-brain synchrony as observed in romantic couples and mother-child interactions. However, there are many more possible relations between participants that have never been tested, for instance, siblings, employer-employee and seller-buyer dyads. In the future, the hyperscanning should explore other relationships between humans and emotions related to them. Second, inter-brain synchrony is higher for scenarios involving empathetic behavior, especially when these include an active emotional component. To sum up, the intensity of the emotional component modulates the synchronous neuronal activity during social interactions. Still, further research needs to be driven on this topic. For instance, the effect of well-studied emotions as stress or disgust must be investigated. This can shed light on whether the impact of negative emotional interactions induces more synchronize behavior than in the presence of emotions of a positive valence or if, instead, the modulatory effect of these might slightly depend on the sort of task.

### Cooperation and Competition

Hyperscanning studies have addressed cooperative and competitive contexts under several methodological paradigms. These allow for the study of both conditions within the same setup. Therefore, participants can either cooperate or compete to achieve their goal, and meanwhile, intra- and inter-brain activity is recorded. These include, for instance, the Prisoner's dilemma task (Babiloni et al., 2007a; De Vico Fallani et al., 2010), chicken's game (Astolfi et al., 2010), time estimation (Cui et al., 2012), turn-based interaction disk games (Liu et al., 2015, 2016b), Jenga (Liu et al., 2016a), or pong-game (Sinha et al., 2016).

Concerning intra-brain activity, hyperscanning studies reveal some commonalities of activation around the prefrontal cortex (PFC). For instance, during a Prisoner's Dilemma task, Babiloni et al. (2007a) found that mPFC is active during all the conditions (i.e., cooperation, defect, and tit-for-tat). In contrast, ACC is only activated when participants defect. In general, the global integration of brain areas was higher under the competitive condition than in cooperation and tit-for-tat. This is in line with findings by Astolfi et al. (2010) in which defect and tit-for-tat conditions obtained higher activity than for the cooperative condition in beta-band EEG recordings. mPFC has been generally related to social interaction supporting the constant activation observed during all conditions. On the other side, ACC has been linked to the theory of mind, indicating that an extra effort is needed to predict the opponent's behavior under competitive interactions. In another scenario, Liu et al. (2015, 2016b) performed a turn-based interaction in a computerized two-person game. Participants took turns to be either a builder or a helper/obstructer partner; brain activity was recorded using fNIRS. They found significant activation in rIFG in builders during the cooperation condition but not when their partners were competing. A similar set-up was used by Liu et al. (2016a) in which a significantly higher activity was found in the obstructors' rIFG area. However, in both studies, no effect was found for helpers; i.e., no “cooperated effect” was revealed. rIFG has been linked to empathy and intention understanding during interpersonal interactions. In this sense, results showed a need for higher empathy when guidance is necessary to achieve a common goal. On the other side, when it comes to hinder other's performance, the understanding of an opponent's intentions plays a crucial role.

On an inter-brain level, the activation in PFC seems to be modulated by the condition and nature of the task. For instance, in the aforementioned setup, Liu et al. (2015, 2016b) found a significant inter-brain synchrony only in builder obstructor pairs. Additionally, Liu et al. (2016a) observed active inter-brain synchrony in the posterior region of the right middle and superior frontal gyrus, particularly Brodmann area 8 (BA8), during cooperative and obstructive interaction (but not in the parallel game and talking condition). Inter-brain synchrony was also observed only during cooperative interaction in the dorsomedial prefrontal cortex (dmPFC), particularly in Brodmann area 9 (BA9). Since participants are performing a joint activity, motor execution has to be synchronized. This is in line with previous findings linking PFC with functions as planning and motor execution. On the contrary, other studies reported stronger synchronized inter-brain activity in cooperative contexts. As shown when De Vico Fallani et al. (2010) and Babiloni et al. (2007a) performed Prisoner's dilemma setups, hyper brain networks in competitive brains have fewer links and have overall higher modularity than in tit-for-tat and cooperative couples. Furthermore, Cui et al. (2012) found increased coherence between signals measured over the right superior frontal cortices between two brains in cooperative and not during competitive behavior. Supporting these findings, Sinha et al. (2016) reported significantly higher inter-brain synchrony between the subjects when they cooperated as compared to the competitive scenario. Additionally, they found that inter-brain synchrony was enhanced considerably when the subjects were physically separated, i.e., they cooperated via an intranet network. This is in contrast with Liu et al.'s (2015, 2016a,b) findings of synchronized activity in dmPFC in competitive contexts. This might be because different set-ups require synchronized activation under different conditions. For instance, a task like the prisoner dilemma needs a higher understanding of other's intentions when participants decide to cooperate.

All in all, hyperscanning studies confirm previous findings on the crucial role of dmPFC in collective behavior. However, the strength of this synchronized activation in dmPFC depends not only on the condition (i.e., cooperation and competition) but also on the specific kind of task as well. For instance, tasks like turn-taking games (e.g., Jenga) that require the prediction of the opponent's actions demand a higher level of the theory of mind processing. On the other hand, tasks like the prisoner's dilemma imply empathy/theory of mind during the cooperative scenarios, and these differences are also reflected in between-brain analysis. With further development of mobile neuroimaging methods, studying cooperative and competitive situations might be possible in more real-life situations. For example, we can imagine using sports games like football or basketball, where players cooperate and compete at the same time with other players. It would be interesting to see whether results from experimental hyperscanning scale to real-life cooperative and competitive situations.

### Games and Decision Making

Overall studies in the field of games and decision making have shown that their neural underpinnings involve a network of regions. They range from the medial frontal cortex (MFC), superior temporal sulcus (STS), and to the temporoparietal junction (TPJ). Throughout the last years of research in the field of interactive decision making in games, a specialization of focus took place, as the first studies focused on areas active in simple games, such as game theory. However, the first investigations to test the neural basis of social interaction used the game theory, as it allows us to define a social situation in which one may lose or profit. Babiloni et al. (2007a) demonstrated that cooperative social interaction activates the reward circuitry. Non-cooperative behavior, in contrast, does not. Their findings suggest a strong activation of the ACC and the cingulate motor area (CMA). The results point out the importance of the ACC, especially for leaders. In their case, it was the person who plays the first card on the deck.

Besides, Babiloni et al. (2006) presented EEG hyperscanning as a new and valid methodology to address the brain activity of a group during real-life social interaction, the “spirit of the group.” Building upon the findings from Babiloni et al. (2006), they addressed social interaction during a game. The aim this time was to measure the neural activity of different brains simultaneously, particularly neural processes generated by social cooperation or competition. The results are similar, and they also provided evidence for the ACC and the CMA to be maximally active (Babiloni et al., 2007b). One other early experiment in the field of decision making was performed by Tomlin (2006). They investigated the impact of personal and impersonal situations by using fMRI hyperscanning. Their findings were in line with the results by Babiloni et al. (2007a), as the dorsal anterior cingulate cortex responded strongly to their set up. Furthermore, cingulate and paracingulate cortices appear to contribute to social cognition and decision-making.

Further, Tomlin (2006) added the possibility that other variables in the social domain may impact outcomes in this area, like the belief in “me” or “not me.” Also, Yun et al. (2008) studied social decision making by using the Ultimatum Game, as the experimental model offers the estimation of e.g., fairness or mind-reading, which has been used before as well (Sanfey, 2003). They also mentioned, as other authors have done, the umbrella term “theory of mind,” showing how wide the topic can be interpreted. Their results suggested high-frequency oscillations in frontocentral regions, indicating that social interaction is closely related to this area. Investigating the effect of gender in cooperative and non-cooperative situations, Cheng et al. (2015) used fNIRS and revealed that task-related coherence in brain activity. This was evident in regions of the frontal cortex, especially when opposite-sex partners are cooperating. The last study to mention here is the one by Zhang et al. (2017), as they provided an overview of research from the last years and focused on another variable deception. In their study, they used fNIRS hyperscanning to measure pairs of participants in a two-person gambling card game simultaneously. Their findings provided higher TPJ activation in deceptive acts compared to honest ones. Further, they assume that STS may play a critical role in spontaneous deception. Decision making in games offers a well-controlled environment to investigate decision making. Future research has to uncover the precise influence of a known and not know partner, and the differentiation between cooperation and competition. Furthermore, influences like facial expression or gestures are worth considering.

### Action Representation and Joint Attention

Whenever we socially interact with others, we have to coordinate our actions with those of our partners precisely. For successful joint action, we need to understand our partner's intentions and combine it with our action plan, always anticipating, attending, and adapting. In this context, joint attention provides the basis for shared awareness of common objects and goals, which is required to join our actions with others effectively. When studying neural mechanisms underlying these cognitive abilities, hyperscanning research provides new opportunities to investigate the intra- and inter-brain effects that accompany joint action. Setups range from pure natural eye-to-eye contact and mutual visual search to more demanding joint musical performance.

Considering mutual gaze as the communicative context in which joint attention is initiated, Hirsch et al. (2017) investigated the neural effects of natural eye-to-eye contact via fNIRS. Comparing “online” interactive eye-to-eye-contact with an “offline” non-interactive eye-to-picture condition, they reported a broad neural network reacting sensitive to interactive mutual gaze: during online eye-to-eye contact, the hemodynamic signals of the left frontal (pre- and supplementary Motor Cortex) the and temporal-parietal regions displayed a higher functional connectivity within brains as well as increased synchronization between brains. This network vastly overlaps with regions associated with language perception and interpretation (i.e., Broca's and Wernicke's regions). Due the this, Hirsch and his team supposed that natural eye-to-eye-contact actively incorporates face-to-language processing. The cross-brain coherence observed in these areas supports this claim, indicating that the rapid online exchange of information between the brains that enables language processing is also communicatively active during mutual gaze.

Further research investigating mutual gaze has used similar experimental paradigms: they observed the brain activity of two subjects interacting in a non-verbal joint attention task (Saito et al., 2010; Lachat et al., 2012; Koike et al., 2016). Here, subjects had to mutually attend target objects either by following the partner's gaze, by self-initiating the common gaze direction, or by following an external cue. In the hyperscanning fMRI study of Saito et al. (2010), during moments of shared attention, paired subjects showed significantly higher inter-brain correlations in the IFG. They therefore concluded that observed inter-brain synchronization in the right IFG facilitates the formation of shared representations, enabling the incorporation of shared intentions by internalizing the other's intentions.

These findings closely relate to the reports of an extended fMRI study by Koike et al. (2016). In this experiment, the research additionally examined the eye-blink synchronization between the subjects, considering them as an index of joint attention. Alternating between mutual gaze and joint attention tasks, dyads displayed increased synchronization of eye-blinks and right IFG activity when they had been previously engaged in a joint attention task. The researchers take this as an indication that the inter-personal neural synchronization through joint attention can be learned, and therefore, be maintained in the social memory. Similar to Saito et al. (2010), the study also reported significant inter-brain synchronization in the right IFG in the context of initiating as well as responding to joint attention. This synchronized activity also correlated positively with enhanced eye-blink synchronization. Importantly, in a video control condition, where participants did not see their partners as a live recording, the right IFG showed no activity. From these results, the study inferred that the right IFG acted as an interface between the self and the other; it is thus thought to coordinate constant shifts between central-executive and default-mode networks, moving attention between oneself and the partner. This fits well with Saito et al. (2010); they associated the synchronized activity of the right IFG with the formation of shared representations between subjects.

Applying dual EEG to compare the neural activity of the socially driven vs. color-driven gaze direction, Lachat et al. (2012) based their research on different brain oscillations. They focused on frequency bands around 10 Hz over parieto-occipital and centro-parietal since this activity is generally associated with social coordination abilities. As previously expected, they found an attenuation of left-hemispheric alpha and mu rhythms by joint attention. This modulatory effect, however, was characteristic for the mutually directed gaze in general, independent of the type of instruction, i.e., whether it was socially or color driven. The researchers interpreted this suppression of the alpha mu rhythm as an indication for an “attention mirroring system,” which allows subjects to orient their attention jointly. The left lateralization of this alpha mu attenuation contradicts previous research, where neural effects of social interaction are predominantly reported in the right hemisphere (Saito et al., 2010; Dumas et al., 2012; Koike et al., 2016; Novembre et al., 2016).

In contrast to these mutual gaze experiments, Szymanski et al. (2017b) compared individual performance with a joint performance during a visual search task. Here, the interaction between subjects was much more natural since verbal, gestural, and tactile communication could be used freely. The researchers tried to relate within- and between-brain neural dynamics to their respective team performance. Indeed, their results indicated that the overall team performance increased with intra- and inter-brain phase synchronization, especially in lower frequencies at frontal sites. Thus, local as well as between-brain phase synchronization were considered as supportive factors for joint attention performance.

Beyond joint attention, the question of how two persons coordinate their actions with one another is subject of hyperscanning paradigms. Following the notion of co-representation (Sebanz et al., 2003), humans form an internal representation of another person's actions through common coding and mirror neuron mechanisms. This representation helps to adjust their actions in favor of a (joint) goal. However, the nature of human interactions is divers; relationships can be symmetric or complementary and emerge spontaneously or be predefined by the type of social situation. The question of how the representation of the self's and other's actions are modulated in these different contexts was the main subject of the studies discussed in the following section.

Ménoret et al. (2014) investigated changes in electrophysiological patterns when we do not only observe an action but also co-act with our partner by performing a complementary task. They found that co-acting led to stronger movement-related beta suppression and more negative movement-related potentials at frontal sides in observers. This implies that co-acting goes along with a more intense representation of the other's action compared to mere observation. Sebanz et al. (2006) led two people to perform a go/nogo task alone or as a pair sitting side by side. Each subject reacted to a different color cue, while a task-irrelevant stimulus pointed to a side either compatible or not compatible with the side on which the participant who was in turn to press the button was seated. Longer reaction times in the incompatible condition and a stronger Nogo P3 component at frontal and central electrodes in the group condition can be interpreted as a consequence of co-representing the partner's actions and the need to suppress own action-tendencies. Both Ménoret's and Sebanz's findings are in line with the concept of co-representation, indicating that observed as well as expected actions activate the according movement-related mechanisms within partners.

The relation between the anticipation of a partner's actions and dynamical entrainment was subject of Novembre et al. (2016). In his paradigm, subjects either familiar or unfamiliar with the partner's notes played a short melody together while tempo instructions were manipulated. Results showed that subjects unfamiliar with their partner's part acted more adaptively. On a neural level, modulations of alpha power at right centro-posterior sides were found: when subjects knew their partner's part, an incongruent tempo between the pianists led to a power increase, while good entrainment (based on congruent tempo instructions) led to an alpha power decrease. This allows for the interpretation that alpha power modulates processes of self-other integration and segregation. While the former is present when the tempo instructions match, the latter is observed when the tempo of the partner must be ignored in order to follow the instructions.

Dumas et al. (2012) aimed at distinguishing correlates of self-other-agency in a hand gesture imitation paradigm. Contrasting analyses across a broad frequency range (0–48 Hz) were used to extract differences between the conditions “not moving and not observing,” “observing gestures passively,” “performing gestures alone,” “induced imitation,” and “spontaneous imitation.” In induced imitation, the roles of model and follower were predefined by the experimenter, whereas they were established by the subjects in the spontaneous imitation condition. In the conditions where subjects performed and observed and performed gestures, a decrease in alpha mu power was observed over sensorimotor areas, including the temporal-parietal junction (TPJ). Hence alpha mu desynchronization might be a marker of action-perception couplings. When subjects were primarily observing the action, passively or as imitators, theta power increased. In the spontaneous condition, gamma was boosted across parietal regions, possibly representing the shared agency. The activation in parietal areas can be seen as a hint endorsing the relevance of TPJ for the agency and social interaction. Dumas et al. (2010) found an increased between brain phase locking in the alpha mu range during spontaneous synchronization.

While Dumas investigated random gestures, Schippers et al. (2010) addressed meaningful gestures used in a charade game. Gesturers, guessers, and control subjects that observed the gestures without guessing took turns in an fMRI scanner. Intending to find correlates of the mirror neuron system and mentalizing system, the researchers calculated the Granger causality between brains. The results supported the relevance of the mirror neuron system for action representation, as the activity in the parietal region (associated with the mirror neuron system) of the gesturer predicted activity in the mirror neuron system and vmPFC (mentalizing system) of the observer. However, the involvement of the vmPFC was both statistically and theoretically less well-funded than the mirror neuron system.

Based on these hyperscanning findings on joint attention and action representation, the relevance of the mirror neuron system and between-brain connectivity in joint action representation gained further interest. This was shown directly by inter-brain coherence (Dumas et al., 2010; Schippers et al., 2010) as well as indirectly utilizing observation-related potentials and oscillatory patterns elicited during joint action contexts (Sebanz et al., 2006; Ménoret et al., 2014). There were also power modulations related to different modes of (joint) action found across a vast range of frequencies and regions, with alpha mu being the most prominent one, perhaps representing action-perception couplings (Dumas et al., 2012; Lachat et al., 2012; Novembre et al., 2016). When it comes to fluently segregating and integrating self- and other-related information during interpersonal coordination of actions, inter-brain synchronization seems to play a pivotal role.

To further validate the proposed hypotheses ascribed to these effects, repeating experiments in combination with different neuroimaging techniques might be useful to overcome the limitations each method has. This would also increase comparability across setups and thus allow for a complete picture and a better interpretation of the findings.

### Over Two Heads

Naturalistic settings are attractive conditions for studying human interaction because, in such settings, interaction occurs without the intervention of the researcher, increasing the ecological validity of the findings. In the last years, researchers have begun to extend hyperscanning research toward multi-subject setups to increase the natural component of social interactions. Early group studies were EEG hyperscanning of four participants playing the Italian card game “Tressette” (Babiloni et al., 2006, 2007b; Astolfi et al., 2010).

There are a variety of reasons for conducting experiments with a multi-subject design with different ideas of making the studied interaction more natural. Social behavior only evolves in the presence of other people, often groups. The presence of other people might enhance individual task performances (Wahn et al., 2018, 2019). In a dyadic setup, interactions might quickly become predictable. Extending the dyadic setup to larger groups may increase the complexity of the interaction due to the actions influencing more individuals generating more possible outcomes. Competition becomes more competitive, and cooperation tasks might become more complex, requiring better interaction from all members of the group. In the context of musical group performances, the structure of a leader and a follower often does no longer exist, playing in an ensemble requires continuous interaction of all members (Babiloni et al., 2011, 2012). In general, the roles of the participants in the interaction become less discrete. This is similar to many social interactions in daily life. Researchers use these properties for two kinds of experimental designs. Some apply findings, conducted from earlier experiments using dyadic design, to a group design to investigate whether these findings still hold under the more natural conditions. Other publications claim that the effects they want to observe can only be present inside a group interaction. Hyperscanning thus allows for investigation of effects that are only present inside large groups like, for example, classrooms, allowing different and new research questions. Such experiments therefore observe social behavior inside a social setting. There thus exist two main categories of current multi-subject hyperscanning research.

Multi-subject hyperscanning experiments can be used to confirm results derived from less complex social situations in a more natural setting. Gevins et al. (2012) generate a measure to distinguish subjects under the influence of alcohol from others by their EEG data. The measure was derived from EEG data, recorded from non-interactive task performance. This measure is then applied to EEG data, simultaneously recorded from each participant of a cocktail party, and still correctly discriminates subjects under the influence of alcohol or placebos. Multi-subject Hyperscanning experiments hence offer a potential method for investigations regarding social behavior.

For other researchers, hyperscanning offers a new opportunity to precisely record human group interactions to investigate social dynamics. Dikker et al. (2017) investigated brain synchrony from a class of 12 high school students over one semester during regular classroom activities. The results suggested that the individuals that are less engaged with the classroom setting show lower brain-to-brain synchrony than the rest of the group. Nozawa et al. (2016) investigated brain synchrony inside 12 groups of four members playing a word chain game under a cooperative condition, reporting frontopolar interpersonal neural synchronization by natural and unstructured verbal communication. Results like these suggest that multi-subject hyperscanning experiments can also be conducted to observe the effects of social interactions directly.

### Speech and Communication

Speech is one of the most crucial aspects of social interactions in humans. The majority of human-human interactions involve verbal communication. Consequently, it is vital to study it with the hyperscanning method to understand the neural underpinnings of verbal communication. The first study that focused on verbal communication compared inter-brain synchrony between face-to-face and back-to-back dialog and monolog situations (Jiang et al., 2012). They found increased inter-brain synchrony between partners in face-to-face dialog but not in the other type of communications. This result suggests that interactive paradigms are required to observe inter-brain synchrony and that hyperscanning is a valid method to measure it. Similarly, greater inter-brain coherence between partners was found in interactive than non-interactive object-naming and description task (Hirsch et al., 2018) as well as for match over mismatch sentences (İşbilir et al., 2016). In another study, Kawasaki et al. (2013) compared the coordination of speech rhythm between human-human and human-machine dyads. Their results, higher between brain synchronization in theta and alpha bands in temporal and lateral-parietal regions, further corroborate that interaction between communicating humans is related to higher inter-brain synchrony. Moreover, when bigger groups (four participants at once) were studied during cooperative communication, frontopolar inter-brain synchronization was found (Nozawa et al., 2016). Inter-brain synchrony and coherence effects could be merely an epiphenomenon of auditory processing. This question was addressed by Pérez et al. (2017). He pointed out that speech-to-brain synchronization is mediated by low-level auditory mechanisms. Of note is the fact that it is the interactive process, however, that plays a crucial role in the inter-brain synchronization. This evidence gives strong support to claim that interaction between participants of a dialog is related to inter-brain synchrony.

Conveying information between interlocutors is a fundamental facet of human communication, especially between teachers and students. Such a scenario was studied by Holper et al. (2013). A correlation analysis between students and teachers showed that in successful educational dialogs, the brain activity of students and teachers synchronizes. As it is first and the only one study focused on the teacher/student inter-brain synchrony, more research is required to understand this phenomenon.

In general, we believe that studying speech and communication requires interaction between participants, and therefore hyperscanning is the best method to understand the neural basis of speech and communication. However, artifacts generated by speech are difficult to remove, and this limitation has to be addressed appropriately.

### Intervention Methods

Intervention methods are especially appealing because properties of the object of investigation are directly manipulated: the activity of specific neural populations in the brain is up- or downregulated by physiological or pharmacological means. This facilitates changes in behavior to distinct neural processes of social interaction. Mu et al. (2016) applied EEG hyperscanning and studied the effects of oxytocin in males on the performance in a reciprocal synchronization task. The task was to synchronize a button press (varying delay in the second range) with the interaction partner or a computer. In contrast, Novembre et al. (2017) applied transcranial alternating current stimulation targeting the motor cortices of participants of each dyad. The authors compared behavioral measures for differences between in-phase and out-of-phase stimulation across subjects in a joint tapping paradigm. Similarly, Szymanski et al. (2017a) targeted the effects of same-phase-same-frequency hyper-tACS on the performance of participants in a joint drumming experiment. All three studies used synchronicity of behavior as a behavioral measure. Significant effects of Oxytocin on the mean alpha-band inter-brain PLV of posterior and central electrodes of males were found only for the social condition. However, most electrodes showed significant differences in this condition. In contrast, if participants synchronized their behavior to a computer, the difference between the treatment group and control was absent (Mu et al., 2016). Results from named tACS-studies show deviating results. Novembre et al. (2017) found higher inter-personal tapping synchrony for in-phase stimulation only for stimulation at 20 Hz. In contrast, Szymanski et al. (2017a) did not find meaningful effects of in-phase stimulation on behavior. Future research may profit from the increase of understanding of intervention methods and theoretical grounding of expected and observed effects. It is challenging to draw a conclusion with only three studies. Therefore, the understanding of inter-brain relations might be fostered by an increased amount of studies applying different intervention methods in combination with hyperscanning.

## Conclusions

Taken together, in this review, we first presented methods that are used to measure the brain activity of two or more participants simultaneously. We discussed their advantages and disadvantages for studying different aspects of social interaction. Further, we reviewed the analysis methods that are used to study between brain networks. We listed different types of analyses that can contribute to various aspects of our understanding of the social brain. In the final section, we presented results of hyperscanning studies performed in the last two decades that focused on diverse cognitive functions and their neural underpinnings.

All these methods, analysis, and experimental results are in line with the call for a more ecologically valid way of studying the social brain (Hari and Kujala, 2009; Hasson et al., 2012; Hari et al., 2013; Schilbach et al., 2013; Redcay and Schilbach, 2019; Konvalinka and Roepstorff, 2012). This call is present since the last decade and suggests that we need more interactive paradigms and neuroimaging data coming from more than one brain to understand the human brain and its social nature fully. Social interactions are a fundamental part of every human being's life, and studying them is indispensable for neuroscience. The previously challenging idea of hyperscanning research was addressed in last years in multiple ways. With our review, we presented an overview and the results of this effort. Taken together, the results of different hyperscanning studies presented support the claim that hyperscanning is a useful and promising method to study social interaction. Inter-brain synchrony appears to be related to the interaction between participants. Without simultaneous measurements of more than one brain, it would not be possible to explore neural underpinnings of social interaction. However, as the field of hyperscanning is young, and in most cases, only exploratory, more research is required to understand all principles and neural basis of human social behavior. Furthermore, presented here results may give rise to a more extended view on studying the human brain. Namely, the fact that brains of participants synchronized with each other may raise a question of whether studying higher cognitive functions should include more participants to understand the human brain fully.

In sum, with the evidence presented in this review, we tried to give an informed overview of the field and point out future avenues of research to foster insights into the interacting mind/brain.

## Author Contributions

AC and PK organized and supervised the reading club at the University of Osnabrück to summarize and discuss hyperscanning literature. AC, SE, AL, DM, MG, SS, FS, ZR, and PK summarized all papers discussed in the review and wrote the review. AC and PK drafted and revised of the manuscript.

### Conflict of Interest

The authors declare that the research was conducted in the absence of any commercial or financial relationships that could be construed as a potential conflict of interest.
